# Long-term efficacy and safety of sirolimus therapy in patients with lymphangioleiomyomatosis

**DOI:** 10.1186/s13023-019-1178-2

**Published:** 2019-08-20

**Authors:** Siqi Hu, Xiuxiu Wu, Wenshuai Xu, Xinlun Tian, Yanli Yang, Shao-Ting Wang, Song Liu, Xingxiang Xu, Kai-Feng Xu

**Affiliations:** 1 Department of Pulmonary and Critical Care Medicine, Peking Union Medical College Hospital, Chinese Academy of Medical Sciences, Peking Union Medical College, Beijing, 100730 China; 2 Department of Pulmonary and Critical Care Medicine, North of Jiangsu People’s Hospital, Yangzhou, 225001 China; 3 Department of Respiratory Medicine, the Second Affiliated Hospital of Xiangya, Central South University, Changsha, 410013 Hunan China; 4 Department of Pulmonary and Critical Care Medicine, Beijing Tiantan Hospital, Capital Medical University, Beijing, 100050 China; 5 Rare Diseases Research Center, Chinese Academy of Medical Sciences, Beijing, China; 6 Central Laboratory, Peking Union Medical College Hospital, Chinese Academy of Medical Sciences, Peking Union Medical College, Beijing, 100730 China

**Keywords:** Lymphangioleiomyomatosis, Sirolimus, Pulmonary function

## Abstract

**Background:**

Sirolimus has been confirmed to be effective for lymphangioleiomyomatosis (LAM), a rare multisystem neoplastic disease in women. The long-term effects of sirolimus treatment for LAM, however, are largely unknown. We aimed to analyze the long-term efficacy and safety of sirolimus therapy for LAM with 4-year follow-up.

**Methods:**

In total, 142 sporadic LAM patients who took sirolimus for 1–4 years were retrospectively enrolled for this analysis. The variables used for analysis included pulmonary function tests, arterial blood gas analysis, 6-min walking distance (6MWD), St. George’s Respiratory Questionnaires (SGRQ) and serum vascular endothelial growth factor-D (VEGF-D) levels before and after the initiation of sirolimus therapy. The rates of change (slope) in those variables were calculated, and adverse events were also analyzed.

**Results:**

In total, 122, 83, 60 and 32 patients out of 142 were followed for 1, 2, 3 and 4 years respectively. Sirolimus treatment improved the change rate in forced expiratory volume in 1 second (FEV_1_) and forced vital capacity (FVC) compared with the data before treatment (FEV_1_, − 10 ± 15 vs. − 178 ± 36 ml/y, *P* <  0.001 and FVC, 54 ± 22 vs.-72 ± 68 ml/y, *P <* 0.05). In comparison to the baseline measurements, significant improvements were observed in FEV_1_ at the first year; FVC at 1–2 years; arterial oxygen levels, 6MWD, and SGRQ at 1–3 years; and VEGF-D at 1–4 years. Overall, all variables stabilized or improved during the 4 years of observation. Adverse events related to sirolimus were mild.

**Conclusion:**

Sirolimus therapy is effective at improving or stabilizing pulmonary function, oxygen levels, exercise capacity, and quality of life in patients with LAM for up to 4 years. VEGF-D is maintained at a lower level for 4 years after treatment. Adverse events related to sirolimus were mild.

## Background

Lymphangioleiomyomatosis (LAM) is a rare multisystem neoplastic disease that is characterized by cystic lung destruction, angiomyolipoma and lymphangioleiomyomas [1, 2]. LAM may occur sporadically, or in adults with tuberous sclerosis complex [1]. Cystic remodeling in the lungs compromises lung function, resulting in progressive dyspnea, and finally respiratory failure [3].

Sirolimus (rapamycin) has been confirmed to be effective for the treatment of LAM [4, 5]. In our previous report, sirolimus was shown to improve lung function, arterial oxygen levels, 6 min walking distance (6MWD), St George Respiratory questionnaire (SGRQ) scores and vascular endothelial growth factor (VEGF-D) levels [6]. However, the long-term effects of sirolimus are unclear. Several studies included data on sirolimus treatment over a 2-years period [7–12]. Taveria-DaSilva et al. [8] reported a study in which 44 patients were treated with sirolimus alone, the changes of predicted values of FEV_1_ and DLCO were − 1.7% ± 0.1% and − 2.2% ± 0.1% before treatment and + 1.7 ± 0.3% and + 0.7% ± 0.3% after treatment (*P* <  0.001) during a mean of 2.8 years follow up time. In the recent study, Taveira-DaSilva et al. [9] evaluated the change of pulmonary function of 25 patients with sirolimus treatment, over a period of 4.5 ± 1.6 years, in which annual changes in forced expiratory volume in 1 s (FEV_1_) and diffusion capacity for carbon monoxide (DLCO) were reduced from − 7.4% ± 1.4% to − 0.3% ± 0.5% (*P* <  0.001) and − 6.4% ± 0.9% to − 0.4% ± 0.5% (*P* <  0.001), respectively. Johnson et al. [10, 11] prospectively observed LAM patients treated with sirolimus over 2 years, the mean change in FEV_1_ ranged from − 7 ± 82 ml/year (*n* = 23) to 11 ± 75 ml/year(*n* = 47). The above studies demonstrated that sirolimus effectively improves the lung function in LAM patients. However, it is still unknown whether sirolimus continuously improves or stabilizes lung function over a longer observation period.

Considering sirolimus is used in LAM patients for a long period of time, whether its efficacy can be maintained is a critical question. Safety is another issue for those patients who take sirolimus for many years. In this study, we analyzed the efficacy and safety of sirolimus for up to 4 years.

## Method

### Study populations

Subjects were from the LAM registry in Peking Union Medical College Hospital (PUMCH), Beijing, China. The diagnosis of LAM was re-evaluated and confirmed according to the recent diagnosis criteria of American Thoracic Society and Japanese Respiratory Society published in 2017 [13]. Subjects were included if the following criteria were met: (1) sirolimus therapy with follow-up data after treatment, and (2) sirolimus therapy with baseline evaluation (within 3 months of sirolimus initiation). The exclusion criteria included the following: (1) patients with tuberous sclerosis complex, and (2) patients with other malignant tumors, and (3) patients who had undergone lung transplantation. Patients with tuberous sclerosis complex were not included because of limited data of this group of patients.

The protocol of this study was approved by the Ethical Committee of PUMCH (S-K709). All subjects included in this study signed informed consent documents.

The indication of sirolimus was primarily based on reduced lung function (FEV1 less than 70% predicted value) or rapidly declining lung function (FEV1 loss over 90 ml per year) [5]. Other indications included chylothorax, chylous ascites, angiomyolipomas or repeated pneumothorax, etc. Dosage and dosage adjustment of sirolimus were based on the judgement of the physicians who treated the patient, which have been described in our previous study [6]. Generally, patients took sirolimus 1 or 2 mg orally once daily. A serum level of 5 to 10 ng/ml sirolimus was considered optimal concentration range. For patients with a serum level > 10 ng/ml or < 5 ng/ml, the dose of sirolimus was adjusted according to the clinical symptoms and adverse events.

### Study design

We carefully collected the annual follow-up records of the enrolled subjects for this retrospective analysis. The follow-up visit data comprised pulmonary function tests, arterial blood gas analysis at rest (on room air), 6MWD, Borg dyspnea index, SGRQ, and VEGF-D. The baseline data were defined as those collected within 3 months of sirolimus initiation. The additional data were categorized as pretreatment and posttreatment data, defined as 1 year (± 3 months) or 2 years (± 3 months) before sirolimus initiation and 1 year (± 3 months), 2 years (± 3 months), 3 years (± 3 months), and 4 years (± 3 months) after sirolimus initiation.

Pulmonary function was measured according to the American Thoracic Society/European Respiratory Society (ATS/ERS) Task Force Standardization of Lung Function Testing [14]. The 6MWD was performed based on ATS guidelines [15]. Borg dyspnea index was assessed at the end of the 6MWD test. Patients completed the SGRQ according to the provided instructions. The pneumothorax and chylothorax were evaluated by chest X-ray or CT. Degree of pulmonary cystic lesions and renal angiomyolipoma size was evaluated by CT. Adverse events were assessed according to the Common Terminology Criteria for Adverse Events (version 3.0). Serum VEGF-D levels were measured with an enzyme-linked immunosorbent assay (Quantikinine Human VEGF-D Immunoassay, R&D Systems).

### Statistical analysis

Normally distributed data are reported as the mean ± SD; data that were not normally distributed are reported as the medians and interquartile ranges (median [25, 75%]). The normality of the data was analyzed by the Kolmogorov-Smirnov test. The unpaired *t*-test or Mann-Whitney *U*-test was used to compare continuous variables. The paired *t*-test was used to compare the baseline data with post-treatment data. All reported *P* values are two-sided. *P* values less than 0.05 were considered statistically significant. We used *R* language V3.5.3 (Microsoft, Washington, USA) to build linearmix-effect model in order to assess the effects of sirolimus therapy. Data analyses were also performed in GraphPad Prism V.7.03 (Graphpad, California, USA) and SPSS V.24 (IBM, New York, USA).

## Results

### Demographics

The baseline characteristics and clinical features of the study participants (*n* = 142) are shown in Table 1. Of 142 subjects enrolled, 122, 83, 60 and 32 patients were followed-up for 1, 2, 3 and 4 years respectively.

**Table 1:** Baseline demographic and clinical features of patients with lymphangioleiomyomatosis

Demographics	Numbers	Percentage or value
Total sample size	142	100%
Age (years)		38 ± 9
Sex		
Female	142	100%
Former smoker	0	0%
Complications		
Renal angiomyolipomas	33/142	23.2%
Pneumothorax	40/142	28.2%
Chylothorax	48/142	33.8%
Chyloperitoneum	16/142	11.3%
CT grading^a^		
I	7/142	4.9%
II	9/142	6.3%
III	119/142	83.8%
Pulmonary function		
FEV_1_ (ml)	114	1622 ± 712
FVC (ml)	114	2760 ± 645
FEV_1_% pred	114	58.5 ± 25
FVC% pred	114	85 ± 20.6
FEV_1_/FVC (%)	114	57.5 ± 17.9
RV% pred	107	161.3 ± 79.3
TLC% pred	107	116.8 ± 103.8
RV/TLC (%)	107	46.25 ± 13.67
DLCO% pred (*N* = 107)	107	40.7 ± 21.2
Arterial blood gas analysis		
PaO_2_ (mmHg)	122	72.3 ± 13.4
P(A-a)O_2_ (mmHg)	114	43.4 ± 51.1
Borg dyspnea index	132	2.5 ± 2.1
6MWD (m)	133	422 ± 113
SGRQ	132	
Symptoms score	132	40.4 ± 24.2
Activity score	132	54.2 ± 24.9
Impacts score	132	40.5 ± 25.8
Total score	132	44.8 ± 23.5
Serum VEGF-D level (pg/ml)	140	3318 ± 2578

Data: mean ± SD

*Abbreviations*: *6MWD* 6-min walking distance, *DLCO* Diffusion capacity for carbon monoxide, *FEV*_1_ Forced expiratory volume in 1 second; *FVC* Forced vital capacity, *PaO*_*2*_ Partial pressure of oxygen; *P(A-a)O*_*2*_ Alveolo-arterial oxygen partial pressure difference, *RV* Residual volume, *SGRQ* St.George’s Respiratory Questionnaire, *TLC* Total lung capacity, *VEGF-D* Vascular endothelial growth factor–D. ^a^: According to the degree of lung involvement, CT was classified as I, II and III grades. Grade I was less than 1/3 of the whole lung field, Grade III was more than 2/3, Grade II was between 1/3 and 2/3 [16]

### Sirolimus improves pulmonary function, oxygen levels, exercise capacity and quality of life

Not surprisingly, in comparison to the pretreatment data, the posttreatment data showed that sirolimus significantly improved pulmonary function (FEV_1_, FEV_1_%predicted, FVC, FVC%predicted, FEV_1_/FVC, DLCO), oxygen levels (PaO_2_, P(A-a)O_2_), 6MWD, SGRQ and VEGF-D levels (Table 2). Over a mean duration of 1.4 ± 0.5 years before the beginning of sirolimus therapy, the FEV_1_ decreased by 178 ± 36 ml per year (7.71% ± 1.20% predicted, *P* <  0.001), and the FVC decreased by − 72 ± 68 ml per year (− 4.11% ± 1.15% predicted, *P* <  0.001). In contrast, over a mean of 2.2 ± 1.1 years of sirolimus therapy, the FEV_1_ changed by − 10 ± 15 ml per year (0.29% ± 0.48%predicted, *P* > 0.05), and the FVC increased by 54 ± 22 ml per year (2.78% ± 0.72%predicted, *P* <  0.001).

**Table 2:** Changes in pulmonary function and other indicators per year before and after sirolimus treatment

Variables	Before treatment		After treatment		Before vs. After
	Mean change per year	^§^*P* value	Mean change per year	^§^*P* value	*P* value
FEV_1_(ml)	-178 ± 36	< 0.001	− 10 ± 15	0.53	< 0.001
FVC (ml)	−72 ± 68	0.29	54 ± 22	0.016	0.017
FEV_1_%pred	−7.71 ± 1.20	< 0.001	0.29 ± 0.48	0.42	< 0.001
FVC%pred	−4.11 ± 1.15	0.009	2.78 ± 0.72	< 0.001	0.008
FEV_1_/FVC (%)	−7.34 ± 1.08	< 0.001	−1.40 ± 0.40	< 0.001	< 0.001
DLCO%pred	−4.12 ± 1.10	0.002	−0.32 ± 0.37	0.40	0.017
PaO_2_ (mmHg)	−5.7 ± 1.3	< 0.001	1.8 ± 0.5	< 0.001	0.002
P(A-a)O_2_ (mmHg)	4.5 ± 1.3	0.002	−1.3 ± 0.5	0.012	0.002
6MWD (m)	−21 ± 6	< 0.001	15 ± 3	< 0.001	< 0.001
SGRQ total score	3.29 ± 2.02	0.11	− 2.65 ± 0.68	< 0.001	< 0.001
VEGF-D (pg/ml)	− 233 ± 185	0.22	− 555 ± 96	< 0.001	< 0.001

Data: mean ± SD. Data were obtained by using mixed-effects models. ^§^*P* was calculated against a slope = 0. ^¶^*P* was calculated by the slope before sirolimus therapy versus the slope after sirolimus therapy

### Pulmonary function changes in patients with chylothorax and those without chylothorax

Forty-eight patients were with chylothorax, and 94 patients were without chylothorax. Pulmonary function data were only available in patients with small amount of pleural effusion. Our data showed that baseline VEGF-D levels were higher in patients with chylothorax, however no significant differences were observed in changes of VEGF-D levels and pulmonary functions in patients with and without chylothorax over 4 years treatment (data not shown). The yearly changes of FEV_1_ in patients with and without chylothorax were 0.46% ± 0.76% predicted and 0.10% ± 0.60 predicted (*P* = 0.95), respectively. The yearly change of DLco were − 0.06% ± 0.69% and − 0.45% ± 0.43% predicted (*P* = 0.92).

### Long-term effects of sirolimus at 1, 2, 3 and 4 years

The main aim of the study was to investigate the long-term effects of sirolimus. Using paired comparison, we were able to detect the differences of the measurements from the baseline to 1, 2, 3 and 4 years after the initiation of treatment. As shown in Table 3, the FEV_1_ improved significantly in the first year, and the FVC improved in the first and second year; then, the significance disappeared during the subsequent follow-up. There were no changes in the diffusion capacity after treatment. Overall, pulmonary function was maintained after the initiation of sirolimus therapy. Sirolimus could potentially stabilize pulmonary function for up to 4 years. The improvements in PaO_2_, P(A-a)O_2_, 6MWD, and SGRQ were maintained for 3 years and disappeared in the fourth year (Table 3). No worsening was observed except for in FEV_1_/FVC during the follow-up period. The decrease in VEGF-D level was maintained for up to 4 years (Table 3). For chylothorax, one patient accepted thoracic duct surgery and sirolimus treatment at the same time and rapidly achieved a complete remission. Among the patients receiving sirolimus treatment without surgery, 35 patients got complete remission, and 12 patients improved.

**Table 3:** Paired comparisons of functional tests and serum VEGF-D levels between the baseline and annual time points during sirolimus treatment

Variable	1 year			2 years			3 years			4 years		
	Baseline	After sirolimus	*P* value	Baseline	After sirolimus	*P* value	Baseline	After sirolimus	*P* value	Baseline	After sirolimus	*P* value
Pulmonary ventilation function	*N* = 67			*N* = 36			*N* = 22			*N* = 11		
FEV_1_ (ml)	1590 ± 690	1640 ± 710	0.045	1640 ± 610	1610 ± 690	0.462	1470 ± 660	1520 ± 740	0.560	1440 ± 590	1310 ± 440	0.099
FVC (ml)	2640 ± 760	2830 ± 700	0.004	2700 ± 620	2880 ± 560	0.044	2680 ± 660	2780 ± 630	0.398	2570 ± 560	2800 ± 380	0.149
FEV_1_%pred	55.97 ± 23.75	59.28 ± 24.3	0.004	57.48 ± 21.97	58.28 ± 24.6	0.705	54.22 ± 23	57.02 ± 26.44	0.346	50.38 ± 21.09	49.19 ± 17.75	0.557
FVC%pred	80.85 ± 22.37	87.87 ± 24.06	0.005	83.2 ± 17.94	89.68 ± 18.03	0.019	83.56 ± 22.27	92.08 ± 22.08	0.054	81.32 ± 18.72	90.84 ± 15.3	0.128
FEV_1_/FVC	56.27 ± 17.42	56.33 ± 18.02	0.966	58.32 ± 20.66	53.72 ± 21.82	0.000	53.59 ± 22.1	52.94 ± 23.07	0.812	54.04 ± 19.9	45.25 ± 15.17	0.014
Gas exchange	*N* = 48			*N* = 26			*N* = 19			*N* = 7		
DLCO%pred	40.62 ± 20.34	41.23 ± 20.42	0.378	46.94 ± 23.87	45.23 ± 22.26	0.197	32.86 ± 19.01	35.64 ± 16.91	0.104	34.46 ± 17.65	30.49 ± 12.71	0.109
Arterial blood gas	*N* = 69			*N* = 39			*N* = 31			*N* = 17		
PaO_2_ (mmHg)	66.99 ± 12.58	71.21 ± 14.98	0.006	68.14 ± 12.61	75.68 ± 16.5	< 0.001	66.34 ± 10.68	70.63 ± 13.36	0.026	69.24 ± 9.662	74.09 ± 11.02	0.084
P(A-a) O_2_ (mmHg)	41.29 ± 13.51	36.67 ± 16.07	0.013	40.6 ± 13.28	32.97 ± 17.23	< 0.001	42.63 ± 12.13	36.76 ± 13.89	0.007	37.89 ± 12.72	35.61 ± 11.6	0.504
6-min walk test	*N* = 67			*N* = 34			*N* = 27			*N* = 12		
6MWD (m)	417 ± 122	461 ± 105	< 0.001	420 ± 120	472 ± 98	< 0.001	408 ± 105	459 ± 107	0.004	379 ± 145	436 ± 96	0.1572
SGRQ	*N* = 63			*N* = 33			*N* = 27			*N* = 12		
Total score	46.11 ± 22.11	38.37 ± 22	< 0.001	42.85 ± 21.83	31.21 ± 21.79	< 0.001	48.04 ± 23.24	38.93 ± 21.94	0.0078	53.25 ± 24.51	49.08 ± 21.95	0.5019
Serum VEGF-D level	*N* = 89			*N* = 50			*N* = 34			*N* = 15		
VEGF-D (pg/ml)	3594 ± 3156	2001 ± 1972	< 0.001	3711 ± 3714	2133 ± 2550	< 0.001	3280 ± 2228	1761 ± 1255	< 0.001	3445 ± 2453	1851 ± 1355	< 0.001

### Dosage of sirolimus

The mean dosage of sirolimus was 1.59 ± 0.50 mg/d (range, 1 to 2 mg/d) at the beginning, and it was 1.27 ± 0.47 mg/d (range, 0.5 to 2 mg/d) after an adjustment period of 1–6 months. Ninety patients accepted the tests of the serum concentration of sirolimus in the first year. The average blood sirolimus level was 7.2 ± 2.6 ng/ml (range, 1.5 to 18.6 ng/ml).

### Frequencies of adverse events

The adverse events that occurred in our study during the observation period are listed in Table 4. In the first year, the most frequent adverse events were mouth ulcer (68.2%), menstrual abnormality (57.9%), acne (34.6%), weakness (11%), diarrhea (6%), and peripheral edema (5.6%). During the next 3 years, the common adverse events were mouth ulcer, menstrual abnormality and acne. However, the incidence rate of mouth ulcer, menstrual abnormality and acne reduced to 23.3, 26.7 and 10% in the fourth year. Occurrence of adverse events could reduce over time (Table 4). We did not observe severe adverse events in our follow-up periods. Three patients required the temporary discontinuation of sirolimus therapy due to menstrual abnormalities, and then restarted the therapy after 3 to 6 months. No other patients had to discontinue sirolimus therapy because of adverse events.

**Table 4:** Adverse events while taking sirolimus according to the duration of treatment in patients with LAM

Time after sirolimus (years)	Number (%)			
	Year 1 (*N* = 107)	Year 2 (*N* = 70)	Year 3 (*N* = 53)	Year 4 (*N* = 30)
Mouth ulcer	73 (68.2)	33 (47.1)	22 (41.5)	7 (23.3)
Menstrual abnormality	62 (57.9)	34 (48.6)	16 (30.2)	8 (26.7)
Acne	37 (34.6)	22 (31.4)	12 (22.6)	3 (10.0)
Ovarian cysts^a^	17/55 (30.9)	10/37 (27.0)	6/25 (24.0)	1/8 (12.5)
Weakness	11 (10)	1 (1.4)	0	0
Peripheral edema	6 (5.6)	2 (2.9)	1 (1.9)	1 (3.3)
Diarrhea	6 (5.6)	1 (1.4)	0	0
Rash	5 (4.7)	0	0	1 (3.3)
Nausea	5 (4.7)	0	1 (1.9)	0
Weight loss	5 (4.7)	0	0	0
Arthralgia	4 (3.7)	0	0	1 (3.3)
Abdominal pain	3 (2.8)	2 (2.9)	0	0
Pruritus	3 (2.8)	1 (1.4)	0	0
Abdominal distention	2 (1.9)	0	0	0
Fever	2 (1.9)	0	0	0
Chest pain	2 (1.9)	1 (1.4)	1 (1.9)	1 (3.3)
Toothache	1 (0.9)	0	0	0
Gingival hyperplasia	1 (0.9)	0	0	0
Periodontitis	1 (0.9)	0	0	0
Herpes zoster	1 (0.9)	1 (1.4)	0	0
Alopecia	1 (0.9)	0	0	0
Headache	1 (0.9)	0	0	0
Dizziness	1 (0.9)	0	0	0
Palpitation	1 (0.9)	0	0	0
Myalgia	1 (0.9)	0	0	0
Tenosynovitis	1 (0.9)	0	0	0

^a^based on patients evaluated

## Discussion

Whether the efficacy of sirolimus can be maintained during long-term treatment is a critical question. We partially answered this question in the present study. We found improvements of sirolimus in the following parameters in patients after beginning sirolimus treatment: the FEV_1_ in the first year; the FVC in the first and second year; arterial oxygen levels, exercise capacity and quality of life in the first, second and third year; and the VEGF-D levels in all 4 years. An encouraging finding was that most measurements improved or stabilized during 4-year observations after sirolimus treatment.

Current clinical trials usually observed 12–24 months of sirolimus treatment. Several studies included observations over 2 years. In studies with 2–4 years observation, the increase in the FEV_1_ varied from 11 ml/year to 50 ml/year after starting sirolimus treatment [6, 7, 10–12]. In a 4-year prospective study, the mean change in the FEV_1_ in patients not receiving sirolimus was -70 ml /year (*n* = 66), while the mean change in patients receiving sirolimus was 7 ml/year (*n* = 23) [10]. Taveira-DaSilva et al. [9] observed 25 patients for 4.5 years, and the FEV_1_ and DLCO changed slightly. The change of FEV_1_ was − 10 ml/year (*P* = 0.53) and the FVC increased by 54 ml/year (*P* = 0.016*)* in our study. In the paired comparison analysis, we found that the FEV_1_ benefited most from sirolimus in the first year, and the FVC benefited in the first 2 years. No significant reduction in pulmonary function was observed over the 4 years. The efficacy of sirolimus can be maintained for at least 4 years.

Higher VEGF-D levels were observed more frequently in patients with lymphatic disease who presented with chylous pleural effusion or ascites than in patients without lymphatic involvement [17]. Several studies reported that compared with patients without lymphatic disease, patients with lymphatic involvement may experience a great improvement in pulmonary function after starting sirolimus therapy [7, 9]. Taveira-DaSilva et al. [9] observed that the effects of sirolimus on VEGF-D levels and DLCO were especially marked in patients with LAM with lymphatic involvement than those without lymphatic involvement. For patients with or without limited small amount of chylothorax, improvement of pulmonary function was similar.

The safety profile is satisfactory. Patients tolerated sirolimus treatment very well. The rate of adverse effects decreased during the follow-up. We also analyzed the group of patients who were followed up for 3 and 4 years, the rate of adverse effects were decreased over time (data not shown).

Anyway, we still need to be cautious in observing long-term adverse effects from the treatment. LAM patients use sirolimus for many years, and it is important to monitor their progress and safety data regularly, at least once a year. Novel therapies for LAM are urgently needed for use when sirolimus cannot be used because of insensitivity or resistance of sirolimus.

The limitations of this study include its retrospective nature and the limited sample size during observation, as only 32 patients had data for all 4 years. Beginning in 2016, a national LAM registry in China was planned that will recruit 800 LAM patients and conduct yearly follow-up. We hope that some unanswered questions will be clarified in future studies.

In conclusion, sirolimus therapy is effective at improving or stabilizing pulmonary function, oxygen levels, exercise capacity, and quality of life in patients with LAM for up to 4 years. VEGF-D maintained at lower level for 4 years after the initiation of treatment. Adverse events related to sirolimus was mild during the follow-up period.

## Data Availability

The datasets used and analysed during the current study area available from the corresponding author.

## Abbreviations

%pred: % predicted
6MWD: 6-min walking distance
ATS: American Thoracic Society
DLCO: Diffusion capacity for carbon monoxide
ERS: European Respiratory Society
FEV_1_: Forced expiratory volume in 1 s
FVC: Forced vital capacity
LAM: Lymphangioleiomyomatosis
P(A-a)O_2_: Alveolar-arterial oxygen gradient
PaO_2_: Partial pressure of oxygen in arterial blood
RV: Residual volume
SGRQ: St. George’s Respiratory Questionnaire
TLC: Total lung capacity
VEGF-D: Vascular endothelial growth factor-D
